# Positive psychological resources and support needs among patients with gastric cancer experiencing fear of cancer recurrence during chemotherapy: a qualitative study

**DOI:** 10.3389/fpsyg.2026.1975541

**Published:** 2026-10-21

**Authors:** Wanjun Zhao, Yumei Huang, Yue Fan, Dan Yu, Yinghui Hu

**Affiliations:** 1 School of Nursing, Jiangxi Medical College, Nanchang University, Nanchang, Jiangxi, China; 2 Department of Nursing, Nanchang Medical College, Nanchang, Jiangxi, China; 3 Jiangxi Provincial People’s Hospital, The First Affiliated Hospital of Nanchang Medical College, Nanchang, Jiangxi, China

**Keywords:** chemotherapy, fear of cancer recurrence, gastric cancer, PERMA model, positive psychology

## Abstract

**Objective:**

To explore positive psychological resources and support needs among patients with gastric cancer undergoing chemotherapy who experience fear of cancer recurrence (FCR), guided by the Positive Emotion, Engagement, Relationships, Meaning, and Accomplishment (PERMA) model.

**Methods:**

A qualitative descriptive study was conducted with 15 patients with gastric cancer undergoing chemotherapy who were recruited using purposive sampling from the Department of Oncology at a tertiary Grade A hospital in Nanchang, China. Data were collected between May and June 2026 through face-to-face semi-structured interviews and analyzed using directed content analysis. The five PERMA dimensions served as *a priori* categories, while open coding was applied to data that could not be accommodated within the framework. The study was reported in accordance with the Consolidated Criteria for Reporting Qualitative Research (COREQ) checklist.

**Results:**

Six categories comprising 17 subcategories were identified: positive emotional resources and psychological reassurance needs; engagement resources and needs for tailored activity participation; relationship resources and interpersonal support needs; meaning reconstruction and reflection on life; accomplishment resources and self-efficacy support needs; and needs for specialized identification and management of FCR.

**Conclusion:**

Patients described multidimensional positive psychological resources across the PERMA domains, together with related support needs and additional FCR-specific needs. These findings suggest that future supportive care programs may benefit from considering patients’ existing psychological resources, support preferences, and FCR-specific needs.

## 1 Introduction

Gastric cancer constitutes a substantial proportion of the global cancer burden, particularly in Asia (Bray et al., 2024). According to the latest GLOBOCAN 2024 estimates, approximately 980,000 new cases and 642,000 deaths from gastric cancer occurred worldwide in 2024, ranking fifth among all malignancies in both the number of new cases and deaths (Sung et al., 2026). Marked sex differences were also observed, with both incidence and mortality rates being substantially higher in men than in women worldwide (Sung et al., 2026). In China, gastric cancer continues to impose a substantial disease burden, ranking sixth in the number of new cancer cases and fourth in the number of cancer deaths, with both incidence and mortality rates generally increasing with age (Zheng et al., 2026). Currently, the treatment of gastric cancer has shifted from surgery alone to a multimodal treatment strategy centered on surgical resection. Chemotherapy is an important component of comprehensive treatment for gastric cancer; however, patients often experience multiple stressors during treatment, including symptom burden (Huang et al., 2025) and role limitations (Yılmaz et al., 2023). Uncertainty regarding treatment efficacy and prognosis may further increase the psychological burden on patients.

Fear of cancer recurrence (FCR) is a common psychological concern among patients with cancer (Luigjes-Huizer et al., 2022). It refers to an individual’ s fear, worry, or concern that cancer may come back or progress and can range in severity from normal concern to clinically significant levels characterized by repetitive thoughts, psychological distress, and functional impairment (Lebel et al., 2016). FCR is closely associated with psychological distress, including anxiety and depression (Podina et al., 2023), as well as reduced quality of life (Shin et al., 2022). Physical discomfort, follow-up appointments, waiting for test results, treatment information, among other factors, may all trigger FCR (Bergerot et al., 2022). Research shows that a substantial proportion of patients with cancer have moderate or severe FCR (Luigjes-Huizer et al., 2022), but routine cancer care still fails to identify this condition adequately; relevant consensus guidelines call for the establishment of screening, referral, and treatment pathways (Hall et al., 2024).

Among patients with gastric cancer undergoing chemotherapy, repeated treatment, physical discomfort and prognostic uncertainty may interact, such that FCR extends beyond fear of disease outcomes to affect patients’ daily lives. Existing research indicates that FCR in patients with gastric cancer is influenced by a combination of physical, psychological, and social factors, and is closely associated with physical symptoms, psychological distress, social support and health-related quality of life (Shin et al., 2022). In patients undergoing chemotherapy for gastric cancer, psychological resilience may mediate the association between social support and FCR (Yang et al., 2024). Recent research has also shown that rumination and catastrophizing mediate the relationship between time perspective and FCR (Wang T. et al., 2025). Beyond the factors and mechanisms identified in quantitative studies, qualitative research has further described the diverse manifestations and specific triggering contexts of FCR, as well as the coexistence of positive and negative coping responses among patients (Zhao et al., 2025). Overall, previous studies have provided preliminary insights into the correlates, psychological mechanisms, and coping characteristics of FCR in patients with cancer, but insufficient attention has been paid to the positive psychological resources that patients possess and their specific support needs.

The PERMA model comprises five dimensions: positive emotion, referring to positive subjective experiences; engagement, involving deep absorption or flow in activities; relationships, reflecting positive connections with others; meaning, involving a sense of purpose, value, and significance in life; and accomplishment, referring to the pursuit and attainment of achievement (Seligman, 2011). Together, these dimensions provide a theoretical framework for exploring patients’ positive psychological resources and related support needs and for structuring the *a priori* categories used in directed content analysis. Previous studies suggest that positive psychological interventions based on the PERMA model may reduce FCR levels among patients with cancer, although the certainty of the evidence remains low (Wang Q. et al., 2025). Designing interventions directly on the basis of theory without adequately understanding patients’ lived experiences may result in a mismatch between the intervention content and their physical condition, life circumstances, and support preferences.

Accordingly, this study aimed to use the PERMA model as a theoretical framework to explore the positive psychological resources and support needs of patients with gastric cancer undergoing chemotherapy who experience fear of cancer recurrence. It also sought to identify professional support needs beyond the scope of the PERMA framework, thereby informing the development of targeted psychological support programs.

## 2 Materials and methods

### 2.1 Study design

This study adopted a qualitative descriptive design informed by the PERMA model to explore the positive psychological resources and support needs of patients with gastric cancer undergoing chemotherapy who experienced fear of cancer recurrence. The PERMA model informed the development of the semi-structured interview guide and provided the *a priori* framework for directed content analysis. Data that could not be accommodated within the predefined framework were subjected to open coding (Hsieh and Shannon, 2005). The study was reported in accordance with the Consolidated Criteria for Reporting Qualitative Research (COREQ) checklist (Tong et al., 2007).

### 2.2 Study setting and recruitment

Patients undergoing chemotherapy for gastric cancer were recruited using purposive sampling from the Department of Oncology of a tertiary Grade A hospital in Nanchang between May and June 2026. Among patients who met the eligibility criteria, variation in disease stage was considered during recruitment to capture experiences across different stages of gastric cancer. The inclusion criteria were as follows: (1) pathologically confirmed gastric cancer; (2) currently receiving chemotherapy and having completed at least one cycle; (3) age ≥18 years; (4) a score of ≥34 on the Chinese version of the Fear of Progression Questionnaire–Short Form (FoP-Q-SF) (Wu et al., 2015); (5) being alert and able to understand the interview questions and communicate verbally; and (6) voluntary participation in the study. A score of ≥34 was used as a screening threshold for clinically significant fear and was not treated as a gastric cancer–specific diagnostic cutoff. The exclusion criteria were as follows: (1) the presence of another primary malignancy or a serious illness that could substantially interfere with the interview; (2) a critical clinical condition that precluded completion of the interview; and (3) lack of awareness of their diagnosis.

During the recruitment period, a total of 29 patients were screened for eligibility. Eligibility was assessed by the nurse manager based on the inclusion and exclusion criteria, with clinical information obtained from medical records and elevated cancer-related concerns screened using the FoP-Q-SF. Potentially eligible patients were approached face-to-face by the nurse manager and initially introduced to the study, after which the first author provided further information about the study, answered any questions, and confirmed their willingness to participate. Of the 29 patients screened, 16 met the eligibility criteria, and 15 were invited to participate. Recruitment ceased after the 15th interview; therefore, the remaining eligible patient was not approached. All 15 invited patients agreed to participate and completed the interviews, and none withdrew.

### 2.3 Data collection

Data were collected through face-to-face semi-structured interviews conducted by the first author, a female registered nurse (RN) who was enrolled in a master’s program and had received systematic training in qualitative research methods and interviewing. She had no prior relationship with the participants. The first and second authors both had nursing backgrounds. The second author had also received training in qualitative research methods, and they were both involved in data coding and analysis. The third author, an experienced nurse manager, assisted with eligibility screening, initial study introduction, interview coordination, and scheduling but did not participate in the interviews or data analysis. Interviews were conducted in quiet, private ward rooms or teaching rooms when participants were in a stable condition and no medical procedures were scheduled. Only the researcher and the participant were present during each interview. The interview guide was developed based on the five dimensions of the PERMA model, refined following discussion with five experts in nursing, oncology nursing, and psychological care, and pilot tested with one patient before the formal interviews to identify any necessary revisions (Supplementary File 1). Before each interview, participants were informed of the study purpose, confidentiality, and their right to withdraw, and written informed consent was obtained. Interviews lasted 20–42 min, were audio-recorded, transcribed verbatim within 24 h, and anonymized using participant codes P1–P15. Brief field notes were made during each interview to document key observations and points requiring clarification. No repeat interviews were conducted. The verbatim transcripts were returned to participants for comment and correction before completion of the analysis.

Data collection and preliminary analysis were conducted concurrently. Saturation was assessed by the two researchers involved in coding through ongoing comparison of the existing codes and subcategories across interviews. Saturation was assessed at the level of codes and subcategories. After the 12th interview, the coding structure had become relatively stable, and interviews 13–15 did not result in substantive changes to the existing codes or subcategories. Saturation was therefore considered to have been reached after the 15th interview.

### 2.4 Data analysis

The interview data were analyzed using directed content analysis, with the five PERMA dimensions serving as *a priori* categories. For coding purposes, positive emotion referred to positive affective experiences such as happiness, relief, reassurance, hope, and similar experiences, together with related support needs; engagement referred to deep immersion or a state of “flow” during activities, which enabled patients to temporarily alleviate illness-related worries, together with related support needs; relationships referred to supportive interpersonal connections and interactions, together with related support needs; meaning referred to reflections on life purpose, values, priorities, and the significance attributed to illness and life experiences, together with related support needs when present; and accomplishment referred to perceived competence, autonomy, goal attainment, and other experiences reflecting personal capability, together with related support needs. Two researchers independently read and reread the original Chinese transcripts, extracted meaning units, and coded the data using Excel. Data not adequately explained by the PERMA framework were retained for open coding and inductively organized into additional categories. When a meaning unit contained analytically distinguishable ideas, it was divided into separate units where feasible. When overlapping meanings could not be meaningfully separated, the unit was assigned to a single primary PERMA category according to the operational definitions and its dominant experiential focus and function within the interview context. Coding disagreements were first discussed by the two coders with reference to the operational definitions and original transcripts; unresolved disagreements were discussed with the senior researchers (the fourth and fifth authors) until consensus was reached. After the preliminary category structure was developed, the research team reviewed the final categories and subcategories for consistency with the original data and for clear differentiation between the five PERMA domains and the FCR-specific category. Any proposed revisions were discussed until consensus was reached. Details of the analytical process are provided in Table 1.

**Table 1:** Steps for directed content analysis.

Steps	Specific operations	Example
Data familiarization	All data were read repeatedly to develop a preliminary understanding of the phenomenon under study, the narrative context, and the overall meaning, while initial analytic memos were recorded.	Participants compared their life priorities before and after illness, reflecting a renewed recognition of the value of their own health.
Establishing the initial categorization framework	Initial categories were determined based on an existing theory or conceptual framework, and the definitions for each category were specified.	Based on the PERMA model, five *a priori* categories, including positive emotion, engagement, relationships, meaning, and accomplishment, were established.
Extracting meaning units	Statements or passages relevant to the research question and conveying a relatively complete meaning were extracted.	“In the past, my main priority was caring for my family and children, whereas now I genuinely realize that my own health must come first.”
Generating initial codes	Meaning units were condensed and summarized while preserving their original meanings.	It was coded as “shifting from family caregiving to prioritizing personal health.”
Assigning codes to a priori categories or applying open coding	Initial codes were compared with the operational definitions of the a priori categories, with matching codes assigned to the corresponding categories and data not adequately explained by the PERMA framework subjected to open coding.	The code above was assigned to the PERMA category of “meaning,” whereas “hoping that healthcare professionals would proactively ask whether the patient was worried about recurrence” was coded as “the need for proactive identification of FCR” during open coding.
Developing subcategories or additional categories	Similarities and differences among related codes were compared to form subcategories, while emergent categories were developed for content that could not be adequately explained by the predefined framework.	Codes such as “prioritizing one’s own health” and “cherishing the present” were integrated into the subcategory “value reconstruction: prioritizing health and appreciating the present,” while the need for proactive FCR assessment, information clarification, and support in developing emotion-regulation skills formed the emergent category “needs for specialized identification and management of FCR.”
Verification and reporting	The researchers returned to the original data and examined the consistency between codes and categories in context, with final interpretations determined through research team discussion.	In light of the context, this statement was interpreted as reflecting a reconfiguration of life values rather than merely a health management practice. The need for specialized support for FCR could not be fully captured by the five dimensions of the PERMA model and therefore warranted separate reporting.

All coding and interpretation were conducted using the original Chinese transcripts. Representative quotations were translated into English by the first author and independently checked against the original Chinese text by the second author; both are native Chinese speakers with experience in academic English writing. Contextual equivalence was prioritized to preserve culturally specific meanings, and semantic disagreements were resolved through discussion.

### 2.5 Ethical considerations

This study was approved by the Medical Ethics Committee of Jiangxi Provincial People’s Hospital [No. Ke Kuai 2026 Nanchang Medical College (001)]. All participants took part voluntarily on the basis of full informed consent and signed an informed consent form. The data were used solely for research purposes. All personally identifiable information was anonymized, and the audio recordings were destroyed upon completion of the study.

### 2.6 Rigor and reflexivity

Before data analysis, the first author reflected on how her nursing background and prior familiarity with the PERMA framework might influence data interpretation. Reflexive notes were maintained throughout the analytical process to document the potential influence of her professional background and theoretical assumptions on the study, as well as the strategies used to address these influences. During data collection, probing questions, paraphrasing for confirmation, and post-interview verification of key points were used to clarify and confirm participants’ intended meanings. However, the final categories and findings were not returned to participants for formal checking. During analysis, the first and second authors independently coded the interview data and continuously compared codes, subcategories, and categories with the original transcripts. Disagreements were resolved through discussion within the research team. Data that did not fit the predefined PERMA framework were retained and analyzed through open coding to avoid forcing the findings into predetermined categories. Representative verbatim quotations were used to support the findings, and Excel-based coding records were retained to ensure the traceability of the analytical process. The study was reported in accordance with the COREQ checklist (Supplementary File 2).

## 3 Results

### 3.1 Characteristics of participants

A total of 15 patients undergoing chemotherapy for gastric cancer were included, and all completed the interviews. Participants ranged in age from 36 to 66 years; 10 were men and 5 were women, and all were married. Most participants had completed junior or senior high school. Participants were employed, retired, engaged in farming, or unemployed; gastric cancer stages ranged from II to IV. The participants’ basic characteristics are presented in Table 2.

**Table 2:** Characteristics of participants.

Participant ID	Gender/age (years)	Education	Employment status	Disease stage	Time since diagnosis (months)	Prior gastric surgery	Disease status	Chemotherapy (cumulative cycles received; current treatment line)	FoP-Q-SF score
P1	Woman/53	Junior high school	Employed	III	4	Yes	No evidence of recurrence	Cycles: 2; Adjuvant	44
P2	Woman/63	High school	Retired	II	7	Yes	No evidence of recurrence	Cycles: 4; Adjuvant	45
P3	Man/51	High school	Unemployed	III	8	Yes	No evidence of recurrence	Cycles: 6; Adjuvant	42
P4	Woman/41	Primary school	Unemployed	III	6	Yes	No evidence of recurrence	Cycles: 5; Adjuvant	42
P5	Man/63	Primary school	Unemployed	IV	17	No	Stable disease	Cycles: 13; First-line	40
P6	Man/66	Junior high school	Farming	III	5	Yes	No evidence of recurrence	Cycles: 3; Adjuvant	37
P7	Man/65	Junior high school	Farming	II	6	Yes	No evidence of recurrence	Cycles: 4; Adjuvant	42
P8	Woman/60	Junior high school	Retired	IV	31	No	Stable disease	Cycles: 20; Second-line	35
P9	Man/54	Primary school	Unemployed	IV	12	No	Stable disease	Cycles: 10; First-line	34
P10	Man/50	High school	Employed	II	8	Yes	No evidence of recurrence	Cycles: 5; Adjuvant	43
P11	Man/46	Junior high school	Unemployed	II	5	Yes	No evidence of recurrence	Cycles: 4; Adjuvant	46
P12	Woman/62	High school	Retired	II	7	Yes	No evidence of recurrence	Cycles: 4; Adjuvant	35
P13	Man/58	Junior high school	Employed	III	9	Yes	No evidence of recurrence	Cycles: 7; Adjuvant	47
P14	Man/45	High school	Employed	III	7	Yes	No evidence of recurrence	Cycles: 5; Adjuvant	45
P15	Man/36	College	Unemployed	IV	6	No	Stable disease	Cycles: 7; First-line	38

Time since diagnosis refers to the interval from diagnosis to the interview.

Chemotherapy cycles refer to the cumulative number of chemotherapy cycles received across all treatment phases and treatment lines from the initiation of chemotherapy to the interview.

### 3.2 Analysis results

Directed content analysis was applied to the 15 interview transcripts, with the five dimensions of the PERMA model—positive emotion, engagement, relationships, meaning, and accomplishment—serving as *a priori* categories. The analysis identified positive psychological resources and associated support needs among patients with gastric cancer who were undergoing chemotherapy and experiencing FCR, yielding 14 subcategories within the five predefined categories. Data that could not be adequately captured within the PERMA framework were analyzed using open coding, which yielded an additional category, “needs for specialized identification and management of FCR,” comprising three subcategories and bringing the final coding structure to six categories and 17 subcategories (Table 3). The findings are presented below according to categories and subcategories and are illustrated with representative verbatim quotations.

**Table 3:** Categories and subcategories.

Categories	Subcategories
Positive emotional resources and psychological reassurance needs	Positive emotions in family interactions and daily life Treatment hope and a sense of control over the illness Limited awareness of positive emotions
Engagement resources and needs for tailored activity participation	Engaging in diverse activities, shifting attention, and maintaining identity Needs for tailored low-burden immersive activities, activity facilitation, and companionship
Relationship resources and interpersonal support needs	Family and spousal support as the core source of support Patient–provider trust and empathic communication Needs for peer and social network support Normal social engagement and preferences for self-directed coping
Meaning reconstruction and reflection on life	Value reconstruction: prioritizing health and appreciating the present Reflections on life and shifts in attitudes toward life and death
Accomplishment resources and self-efficacy support needs	Recognition of personal capabilities and maintenance of everyday autonomy Resources for active participation and support needs for achievable goals Ways of recognizing accomplishment and needs for individualized feedback
Needs for specialized identification and management of FCR	Needs for clear explanations of disease and treatment information Needs for proactive identification, attentive listening, and support with simple emotion-regulation skills Needs for home-based self-management and guidance on seeking medical care

#### 3.2.1 Category 1 (P): positive emotional resources and psychological reassurance needs

##### 3.2.1.1 Positive emotions in family interactions and daily life

Patients’ positive emotions arose from interactions with family members as well as everyday experiences of comfort, such as symptom relief, spending time in the sunshine, and taking walks.

“I have three granddaughters and three grandsons, and I feel so happy whenever they video-call me.” (P7)

“Waking up without feeling weak or having my heart race—that’s something genuinely positive for me.” (P15)

“Get out in the sun more, take a walk, and loosen up a bit. If you lie down all the time, your whole body gets stiff. Moving around might even ease some of the discomfort from chemotherapy. It’s much better than lying in the ward and letting your mind run wild.” (P1)

##### 3.2.1.2 Treatment hope and a sense of control over the illness

Improvements in clinical indicators, advances in treatment methods, and clear explanations from healthcare professionals regarding the disease and its treatment helped foster patients’ hope.

“I got my test results a few days ago. The doctor said all my numbers were better than last time and that I was recovering pretty well.” (P3)

“Whenever I ask the doctors or nurses about my condition, they explain everything clearly and tell me not to overthink it. They say staying relaxed is better for my recovery.” (P5)

“When I hear about a promising treatment for stomach cancer, I feel a bit more hopeful.” (P6)

##### 3.2.1.3 Limited awareness of positive emotions

Some participants had difficulty identifying recent positive experiences and reported paying little deliberate attention to such experiences.

“Nothing much has made me happy lately, and I haven’t really paid much attention to that sort of thing.” (P4)

“I have never thought about that.” (P7)

#### 3.2.2 Category 2 (E): engagement resources and needs for tailored activity participation

##### 3.2.2.1 Engaging in diverse activities, shifting attention, and maintaining identity

Participants described shifting their attention away from illness-related thoughts through activities such as watching short videos, spending time in nature, and engaging in activities with family members. These activities also allowed them to reconnect with roles and identities beyond illness.

“Going for a walk and looking at the scenery outside helps me forget about the illness for a while and feel more at peace.” (P6)

“Sometimes I scroll through TikTok. It takes my mind off things a little.” (P12)

“Does building LEGO with my son count? When he puts something together the wrong way, he frowns, takes it apart, and starts all over again. When he gets it right, he holds it up to show it off to me. I just sit beside him and quietly watch. Sometimes he asks me to pass him a piece, and I reach over and hand it to him. In those moments, I feel like I’m simply a dad spending time with my son, not a patient lying in a hospital bed. I feel completely at peace.” (P15)

##### 3.2.2.2 Needs for tailored low-burden immersive activities, activity facilitation, and companionship

Owing to limitations in their physical condition and energy levels, patients preferred simple, accessible, and minimally strenuous activities and wished to receive the necessary materials, practical demonstrations, or companionship.

“I just want to do something simple and relaxing that does not require too much thought, such as embroidering a very simple flower, listening to music, or humming along.” (P1)

“I’m not really sure what activities would be good for me. It would be nice if someone could get everything ready and show me how to do it.” (P2)

“I would certainly prefer a group activity, with someone there to participate alongside me.” (P12)

#### 3.2.3 Category 3 (R): relationship resources and interpersonal support needs

##### 3.2.3.1 Family and spousal support as the core source of support

Family members and spouses were the most consistent sources of support for patients, providing emotional encouragement, assistance with daily activities, support with medical appointments, financial support and constant companionship. This practical and ongoing support alleviated the practical burdens patients faced in their treatment and daily lives and enhanced their sense of being cared for and their sense of security.

“My relatives and friends are all very supportive, but my family’s support means the most to me. They call me every few days to check in. They tell me to focus on getting treatment, not to worry too much, and to stay positive because I’ll get better. They also keep reminding me to eat a little more.” (P1)

“My wife takes care of everything in my daily life, such as bringing me drinks, cooking, and doing the laundry, and she never complains.” (P3)

“My daughter, definitely. She handles everything for me and supports me both financially and in practical ways.” (P10)

##### 3.2.3.2 Patient–provider trust and empathic communication

Clear explanations of examination results, responses to patients’ questions, and understanding of their emotions by healthcare professionals reduced uncertainty and enhanced patients’ psychological sense of security regarding treatment.

“I feel that the doctor and I are on the same team; he patiently explains which test results are better and which still require observation, and I can tell that he sincerely wants me to recover and is actively working out the best treatment plan.” (P4)

“A few sincere words and a little concern from the doctors and nurses are enough to reassure me for a long time.” (P1)

##### 3.2.3.3 Needs for peer and social network support

Patients hoped that interactions with fellow patients and friends would allow them to share experiences and receive emotional support and companionship in their daily lives, thereby alleviating their anxiety and feelings of loneliness.

“When there is nothing else to do, I can chat with other patients; when more people talk together, we do not dwell on things so much, and everyone feels more at ease.” (P1)

“I still want my friends to care about me, support me, and visit or stay in touch more often.” (P11)

“If there were other patients who’d been through the same thing, it would be nice to have a chat with them—just talk about how we pass the time and how we cope with the worry, without having to hold anything back.” (P15)

##### 3.2.3.4 Normal social engagement and preferences for self-directed coping

Patients hoped that relatives and friends would interact with them as they had before the illness, without becoming distant or singling them out because of their cancer. Meanwhile, preferences for emotional disclosure, companionship, and solitude varied among patients, with some preferring to process negative emotions on their own.

“Other people may try to comfort you, but it does not really help; ultimately, you still have to rely on yourself.” (P8)

“Actually, I tend to process these negative feelings on my own.” (P9)

“Of course, I hope to interact normally with my neighbors, friends, and colleagues so that I do not feel like a patient.” (P14)

#### 3.2.4 Category 4 (M): meaning reconstruction and reflection on life

##### 3.2.4.1 Value reconstruction: prioritizing health and appreciating the present

The experiences of illness and treatment prompted patients to reconsider their priorities, shifting their focus from work and family responsibilities toward their own health and encouraging them to devote their limited energy to living well in the present and pursuing desired activities without delay.

“I used to think that there was plenty of time ahead and that nothing really mattered, but after undergoing surgery and chemotherapy, I realized how important health is. Now I take each day as it comes and try to live every day well.” (P3)

“In the past, my main priority was caring for my family and children, whereas now I genuinely realize that my own health must come first.” (P4)

“Life should be lived to the fullest, so whatever you want to do, you should do it while you can rather than always waiting.” (P10)

“Now I feel that being happy every day is what matters most.” (P13)

##### 3.2.4.2 Reflections on life and shifts in attitudes toward life and death

The experiences of illness and treatment prompted some patients to reconsider the finite nature of life. Some patients no longer dwelt excessively on trivial matters and adopted a more accepting attitude toward life and death, whereas others reflected on their lives before illness and expressed regret about not having appreciated their health and life sooner.

“I don’t take things so seriously anymore. I don’t get caught up in little things, and I’ve learned to let things go.” (P6)

“I’m much more at peace with life and death than I used to be.” (P9)

“I didn’t take good enough care of myself before, but there’s no going back now. Regretting it won’t change anything.” (P15)

#### 3.2.5 Category 5 (a): accomplishment resources and self-efficacy support needs

##### 3.2.5.1 Recognition of personal capabilities and maintenance of everyday autonomy

By independently managing medical visits, household tasks, work, and other daily activities, patients affirmed that they remained capable of solving problems and managing their lives. Despite their illness, patients sought to maintain their pre-illness family and occupational roles and preserve a familiar rhythm of daily life, thereby gaining a sense of competence and accomplishment.

“I do whatever I can by myself. When I came to the hospital, my son offered to bring me, but I told him there was no need. I can register and get all the tests done on my own.” (P2)

“As for things at home, I still do whatever I am able to do, just as I did before. I don’t want people to act as if being ill means I can’t do anything anymore.” (P9)

“I work as a security guard at a university and still go to work as usual rather than stopping because of my illness. In this respect, I think I have done quite well.” (P10)

##### 3.2.5.2 Resources for active participation and support needs for achievable goals

Some patients participated actively in treatment and recovery by listening to and recording medical advice and learning self-monitoring methods, demonstrating their capacity to manage their own health proactively. Patients expressed a need for healthcare professionals to collaborate with them in setting concrete, manageable, and attainable daily goals tailored to their current physical capacity, providing clearer direction for action and reinforcing a sense of achievement.

“It would be even better if you had time to help me set small, achievable goals that were not too difficult, such as drinking five glasses of water today.” (P1)

“When the doctor explains what I need to pay attention to, I now listen carefully and take notes, whereas previously I could not remember anything, so I know how to protect myself and monitor my body. I am not passively receiving treatment; I am actively living for myself.” (P4)

##### 3.2.5.3 Ways of recognizing accomplishment and needs for individualized feedback

Patients differed in how they recognized their accomplishments and affirmed their personal value. Some patients affirmed their value by completing tasks or perceiving personal progress, whereas others gained confidence from external affirmation; some participants placed greater importance on self-evaluation, while others found it difficult to identify a sense of accomplishment during treatment and recovery.

“These achievements remind me that I am more than just a person being treated for an illness and that I remain a person of value.” (P2)

“It is difficult to identify anything in particular, because I do not feel that I have done anything especially impressive; I have just carried on with treatment.” (P6)

“When you praise me a little more, I feel that I am doing quite well and become more confident.” (P7)

“It is enough for me to know about my own progress.” (P15)

#### 3.2.6 Category 6: needs for specialized identification and management of FCR

##### 3.2.6.1 Needs for clear explanations of disease and treatment information

Participants wished to understand their examination results, signs of recurrence or disease progression, chemotherapy-related adverse effects, and other relevant information, and expected healthcare professionals to provide explanations in accessible language and respond promptly to questions about physical changes and treatment.

“When the follow-up examination is completed and the results are available, I would like them to explain everything to me more clearly.” (P2)

“I hope they can respond promptly to my questions about my physical condition. For example, if I develop a new side effect, I may not realize that it is treatment-related and might think that something else is wrong.” (P14)

##### 3.2.6.2 Needs for proactive identification, attentive listening, and support with simple emotion-regulation skills

Patients reported that healthcare professionals rarely asked proactively about FCR and expressed a need for them to initiate conversations about such concerns, listen patiently, and teach simple emotion-regulation strategies that patients could use at home.

“But when it comes to the emotional issue of being afraid of recurrence, very few people specifically offer help.” (P1)

“I hope they can ask me proactively whether I have been worrying frequently about recurrence, be willing to listen to me, and not think that I am simply imagining things.” (P2)

“They could teach us some regulation methods so that we can use them at home whenever we start thinking about recurrence.” (P7)

##### 3.2.6.3 Needs for home-based self-management and guidance on seeking medical care

Participants expected to receive specific guidance after discharge regarding daily self-management, self-monitoring, and indications for seeking medical care, enabling them to recognize abnormalities and take appropriate action at home.

“They should tell us what to do after we’re discharged—how to look after ourselves day to day, what signs might mean the cancer has come back or that something isn’t right, and when we need to go back to hospital.” (P12)

## 4 Discussion

Using the PERMA model as the analytical framework, this study identified positive psychological resources and related support needs across the five PERMA dimensions, alongside additional FCR-specific support needs that were not fully encompassed by the framework.

Positive emotions arose from family interactions and ordinary daily experiences, including symptom relief, time outdoors, and walking. These findings suggest that relatively simple and accessible experiences in everyday life may provide meaningful sources of positive emotion during chemotherapy. Participants identified improvements in clinical indicators and clear explanations from healthcare professionals as sources of treatment hope and perceived disease control. These accounts may indicate that uncertainty about bodily changes contributes to FCR-related concerns during chemotherapy. Previous studies suggest that physical discomfort, waiting for examinations and their results, and other cancer-related cues may trigger FCR (Almeida et al., 2019; Fardell et al., 2016). Taken together, the participant accounts and prior evidence suggest that symptom management and timely, clear explanations of bodily changes and examination results may represent relevant components of supportive care for patients experiencing FCR. In addition, some patients in this study had difficulty identifying recent positive experiences and reported paying little deliberate attention to such experiences, suggesting limited awareness of positive emotional experiences. However, the present data do not clarify the reasons underlying this limited awareness. Importantly, participants did not explicitly express a need for guidance in recognizing positive emotional experiences. Nevertheless, for patients who are receptive to such support, encouraging greater attention to positive experiences in everyday life may be considered a potential supportive approach.

Participants described engagement in specific activities as temporarily shifting attention away from illness-related thoughts and bringing identities such as fatherhood and a sense of being an ordinary person back to the foreground. This suggests that the value of activity engagement may extend beyond diverting attention to helping patients reconnect with identities beyond illness. This is consistent with previous studies showing that meaningful activities can help people with cancer shift attention away from cancer and maintain a sense of identity and normality (Adam et al., 2023), while also reinforcing identities beyond cancer (Evans et al., 2026). Previous research has identified normality, autonomy, and being involved as important value themes for patients with cancer (Mitchell et al., 2020). Activity engagement may provide a means of supporting all three values simultaneously. Healthcare professionals may therefore consider offering low-burden activities that align with patients’ physical abilities, interests, and preferences, not simply as a way to pass the time, but as an opportunity to step away from illness-related concerns and reconnect with valued aspects of themselves.

Participants described family members and spouses as their principal relationship resources, providing practical care, emotional companionship, and ongoing support, while healthcare professionals provided informational and emotional reassurance through clear and empathic communication. Previous research has found that both social support and psychological resilience are negatively associated with FCR (Yang et al., 2024). Our findings further indicate that the value of relational support may depend not merely on its extent, but also on the congruence between the form of support and the patient’s individual preferences. In our interviews, some patients needed attentive listening, companionship, and practical assistance, whereas others wished to maintain normal social interactions, preserve space for autonomy or more self-directed coping. These differences suggest that forms of support perceived as helpful by some patients may be less helpful to others. This pattern underscores the need to tailor relational support to individual preferences, consistent with previous research emphasizing supportive care that is congruent with patients’ needs, preferences, and priorities (Kim et al., 2022). Patient-centered care research similarly underscores the importance of individualizing support according to each patient’s values, needs, and preferences (Mitchell et al., 2020). Accordingly, assessment by healthcare professionals may extend beyond the availability of supportive relationships to include patients’ preferred forms of support, while families may be encouraged to adjust emotional and practical assistance according to patients’ expressed needs and preferred level of autonomy.

Illness and treatment prompted some patients to reorder the relative importance of health, family, and work; however, meaning reconstruction encompassed not only appreciation of the present and greater acceptance but also regret, suggesting that meaning-making is not a unidirectional process from negativity to positivity. A systematic review showed that meaning in life and meaning-making may be associated with posttraumatic growth; however, the available evidence is insufficient to support a direct causal relationship between either of these constructs and posttraumatic growth (Almeida et al., 2022). Against this background, patients’ ability to reconsider their life priorities does not necessarily mean that they have achieved positive growth; similarly, regret should not be interpreted as a failure to adapt. Previous literature has similarly cautioned against assuming uniformly positive adaptation following cancer (Menger et al., 2020). Accordingly, healthcare professionals should respect patients’ differing interpretations of their illness experiences and allow for diverse forms of meaning-making without assuming that positive growth is a necessary outcome.

Regarding accomplishment, participants’ accounts suggest that a sense of achievement during chemotherapy may not necessarily arise from major achievements, but from maintaining everyday autonomy and preserving a sense of competence despite treatment-related disruptions. Previous research has likewise highlighted that maintaining usual daily activities and roles can help people with cancer preserve a sense of functioning, identity, control, and independence (van Dongen et al., 2020). Their preference for concrete and attainable goals suggests that manageable tasks may help reinforce a sense of achievement during treatment. Recent research has similarly highlighted the value of short-term, individualized functional goals that are responsive to patients’ functional status (Maddocks et al., 2025). Participants also differed in how they recognized accomplishment, suggesting that perceptions of achievement may vary across individuals. A scoping review showed that evaluation of progress or achievement was frequently incorporated into the follow-up phase of collaborative goal-setting practices in cancer rehabilitation (Ikeuchi et al., 2024). Accordingly, setting attainable goals and acknowledging differences in how patients recognize accomplishment may be important considerations in supportive care during chemotherapy.

Notably, patients not only wanted healthcare professionals to clearly explain examination results, signs that might indicate recurrence, and treatment-related physical changes, but also expected them to proactively inquire about FCR, listen patiently, and provide practical emotion-regulation strategies, while offering clear guidance on self-monitoring, daily self-management, and when to seek medical care after discharge. For some FCR-related needs among patients with gastric cancer undergoing chemotherapy, general positive psychological resources such as positive emotions and social support are not sufficient. These needs also call for specialized identification and management of FCR. Relevant consensus statement emphasized that FCR should not be treated simply as general anxiety or psychological distress and highlighted the need to establish dedicated clinical management pathways for FCR, including screening, referral, and treatment (Hall et al., 2024). Building on this, the FCR clinical management pathway developed by Smith further supports routine screening and triage, as well as stepped care according to FCR severity; however, consensus has not yet been reached on some specific aspects of the timing of screening and triage (Smith et al., 2026). In this context, participants’ expressed needs for clear explanations of test results, proactive inquiry about FCR, and discharge-related guidance suggest that communication of test results and transitions to discharge may provide practical opportunities for healthcare professionals to initiate FCR-related conversations. However, whether these encounters are appropriate as standardized time points for FCR screening requires further investigation.

In summary, patients with gastric cancer undergoing chemotherapy who experience FCR do not simply lack positive psychological resources; rather, in the context of symptom burden, prognostic uncertainty, and role restrictions, they appear to draw on specific bodily experiences, activities, supportive relationships, meaning reconstruction, and affirmation of their capabilities in efforts to maintain or regain a sense of control, normality, and agency. These findings suggest that supportive care may need to combine support for PERMA-related resources with FCR-specific management, tailored to patients’ physical condition and support preferences.

### 4.1 Strengths and limitations

This study utilized the PERMA model as an *a priori* framework and applied open coding to data outside this framework, thereby systematically presenting the positive psychological resources available to patients while identifying the need for specialized FCR management. This clarified the distinction between support that builds on positive psychological resources and clinical FCR management. By presenting the resources patients already possess and the support they expect to receive, this study provides patient-informed directions for future intervention development.

Several limitations should be noted. This single-center study involved a small and relatively homogeneous sample from one tertiary hospital in Nanchang; all participants were married and most had similar educational backgrounds, limiting marital-status diversity and geographical and sociocultural representation and potentially reducing transferability. Participants unable to communicate verbally or unaware of their diagnosis were not represented, and their experiences may differ from those captured here. Although open coding and researcher reflexivity were used, the use of PERMA as an *a priori* framework may still have influenced coding and interpretation. Interviews conducted during active hospital-based chemotherapy may also have been affected by treatment-related symptoms and the clinical environment. Finally, given the conceptual overlap between FCR and fear of disease progression, participants’ accounts may have reflected elements of both concerns.

### 4.2 Implications for clinical practice and future research

The findings of this study provide directions for future intervention research. Future studies could further explore how PERMA-based resource support can be integrated with specialized FCR management and use this approach to develop comprehensive psychological support programs. The feasibility of these programs and their effects on FCR and related psychological outcomes should then be evaluated. Program development should also consider potential differences across disease stages and individual support preferences. Multicenter and longitudinal studies may help further validate and refine the intervention content. The application of stepped care in this population may be considered a preliminary hypothesis for future research and should be further examined through standardized protocol development and prospective evaluation.

## 5 Conclusion

This study highlights the coexistence of multidimensional positive psychological resources and related support needs among patients with gastric cancer undergoing chemotherapy who experience FCR, together with additional FCR-specific needs extending beyond the PERMA framework. The PERMA framework provided a structure for organizing patients’ perceived psychological resources and related support needs, while the additional FCR-specific category identified needs for specialized identification and management of FCR. These findings suggest that future supportive care programs may benefit from considering patients’ existing psychological resources, support preferences, and FCR-specific needs.

## Data Availability

The qualitative data generated during this study are not publicly available because they contain sensitive participant information and are subject to ethical and privacy restrictions. Requests to access de-identified data should be directed to Wanjun Zhao (1262416348@qq.com) and will be considered in accordance with the ethics approval and applicable privacy requirements.

## Supplementary material

SUPPLEMENTARY FILE 1 Semi-structured interview guide.

SUPPLEMENTARY FILE 2 COREQ checklist.
