# Supplementary material for: Positive psychological resources and support needs among patients with gastric cancer experiencing fear of cancer recurrence during chemotherapy: a qualitative study
Source: Front Psychol. 2026 Oct 21;17:1975541. doi: 10.3389/fpsyg.2026.1975541 (PMC13730911; doi:10.3389/fpsyg.2026.1975541)
Supplement: Supplementary file 1 [file Supplementary_file_1.docx]

Supplementary File 1

A pilot interview was conducted with one patient prior to the formal interviews to test the reasonableness of the interview guide and make necessary revisions. The final interview guide is as follows:

①During your treatment, what kinds of things have made you feel happy or relaxed ?

②In what ways would you like to gain more positive feelings ?

③What helps you temporarily take your mind off the worries associated with being ill ?

④What kind of immersive activities would you be willing to engage in, and what assistance would you require ?

⑤Whose support has been most important to you in coping with the illness ?

⑥What support would you hope to receive in terms of interpersonal relationships ?

⑦How has this illness changed your views on life or living ?

⑧During the treatment process, has there been anything that made you feel good about yourself ?

⑨In terms of managing your fear of cancer recurrence, what kind of help would you like healthcare professionals to provide ?
