# Supplementary material for: Positive psychological resources and support needs among patients with gastric cancer experiencing fear of cancer recurrence during chemotherapy: a qualitative study
Source: Front Psychol. 2026 Oct 21;17:1975541. doi: 10.3389/fpsyg.2026.1975541 (PMC13730911; doi:10.3389/fpsyg.2026.1975541)
Supplement: Supplementary file 2 [file Supplementary_file_2.docx]

Supplementary File 2

**The Consolidated Criteria for Reporting Qualitative Research (COREQ)**

| **No.** | **Item** | **Guide questions/description** | **Answer** |
| --- | --- | --- | --- |
| **Domain 1: Research team and reflexivity** | | | |
| Personal Characteristics | | | |
| 1. | Interviewer/facilitator | Which author/s conducted the interview or focus group? | The first author. |
| 2. | Credentials | What were the researcher's credentials? *E.g. PhD, MD* | Registered Nurse ; master's student. |
| 3. | Occupation | What was their occupation at the time of the study? | Master’s student enrolled in a graduate nursing program. |
| 4. | Gender | Was the researcher male or female? | Female. |
| 5. | Experience and training | What experience or training did the researcher have? | Systematic training in qualitative research methods and interviewing. |
| Relationship with participants | | | |
| 6. | Relationship established | Was a relationship established prior to study commencement? | No prior relationship had been established with the participants. |
| 7. | Participant knowledge of the interviewer | What did the participants know about the researcher? e*.g. personal goals, reasons for doing the research* | Participants were informed of the study purpose before the interview. |
| 8. | Interviewer characteristics | What characteristics were reported about the interviewer/facilitator? e.g. *Bias, assumptions, reasons and interests in the research topic* | The first author reflected on the potential influence of her professional background and the assumptions of the PERMA model and maintained reflexive notes throughout the analysis. |
| **Domain 2: study design** | | | |
| Theoretical framework | | | |
| 9. | Methodological orientation and Theory | What methodological orientation was stated to underpin the study? *e.g. grounded theory, discourse analysis, ethnography, phenomenology, content analysis* | Descriptive qualitative design using directed content analysis, guided by the PERMA model, with open coding for data outside the predefined framework. |
| Participant selection | | | |
| 10. | Sampling | How were participants selected? *e.g. purposive, convenience, consecutive, snowball* | Purposive sampling. |
| 11. | Method of approach | How were participants approached? e*.g. face-to-face, telephone, mail, email* | Face-to-face. |
| 12. | Sample size | How many participants were in the study? | 15 participants. |
| 13. | Non-participation | How many people refused to participate or dropped out? Reasons? | All agreed to participate and none withdrew. |
| Setting | | | |
| 14. | Setting of data collection | Where was the data collected? e*.g. home, clinic, workplace* | Relatively quiet, private ward rooms or teaching rooms in the Department of Oncology of a tertiary Grade A hospital in Nanchang. |
| 15. | Presence of non-participants | Was anyone else present besides the participants and researchers? | No. |
| 16. | Description of sample | What are the important characteristics of the sample? *e.g. demographic data, date* | For further details, see Section 3.1 and Table 2 of the manuscript. |
| Data collection | | | |
| 17. | Interview guide | Were questions, prompts, guides provided by the authors? Was it pilot tested? | Yes. |
| 18. | Repeat interviews | Were repeat interviews carried out? If yes, how many? | No. |
| 19. | Audio/visual recording | Did the research use audio or visual recording to collect the data? | Audio recording. |
| 20. | Field notes | Were field notes made during and/or after the interview or focus group? | Yes, during the interview. |
| 21. | Duration | What was the duration of the interviews or focus group? | 20-42 minutes. |
| 22. | Data saturation | Was data saturation discussed? | Yes. After the 12th interview, the coding structure had become relatively stable, and interviews 13-15 did not result in substantive changes to the existing codes or subcategories. Saturation was considered to have been reached after the 15th interview. |
| 23. | Transcripts returned | Were transcripts returned to participants for comment and/or correction? | Yes. |
| **Domain 3: analysis and findings** | | | |
| Data analysis | | | |
| 24. | Number of data coders | How many data coders coded the data? | Two. |
| 25. | Description of the coding tree | Did authors provide a description of the coding tree? | Analysis process, final six-category structure and Tables 1 and 3. |
| 26. | Derivation of themes | Were themes identified in advance or derived from the data? | Both. |
| 27. | Software | What software, if applicable, was used to manage the data? | Microsoft Excel. |
| 28. | Participant checking | Did participants provide feedback on the findings? | No. |
| Reporting | | | |
| 29. | Quotations presented | Were participant quotations presented to illustrate the themes/findings? Was each quotation identified? e*.g. participant number* | Yes. |
| 30. | Data and findings consistent | Was there consistency between the data presented and the findings? | Yes. Findings narrative, representative quotations and Table 3. |
| 31. | Clarity of major themes | Were major themes clearly presented in the findings? | Yes. Six categories clearly presented. |
| 32. | Clarity of minor themes | Is there a description of diverse cases or discussion of minor themes? | Yes. 17 subcategories and variation in participants' accounts. |
